# Link Between Irrational Beliefs and Important Markers of Mental Health in a German Sample of Athletes: Differences Between Gender, Sport-Type, and Performance Level

**DOI:** 10.3389/fpsyg.2022.918329

**Published:** 2022-07-22

**Authors:** Alena Michel-Kröhler, Martin J. Turner

**Affiliations:** ^1^Department of Clinical Psychology and Neuropsychology, Institute for Psychology, Johannes Gutenberg-University Mainz, Mainz, Germany; ^2^Department of Psychology, Faculty of Health and Education, Manchester Metropolitan University, Manchester, United Kingdom

**Keywords:** irrational beliefs, athletes, mental health, anxiety, perfectionism, REBT, population-based differences, scale development

## Abstract

In this article, we conducted the first meaningful study of irrational beliefs (IBs) in a German sample of athletes. Moreover, we investigated associations between IBs and potential general as well as sport-specific markers of mental health in German athletes. As general markers, we considered psychological distress and wellbeing in addition to IBs, and as sport-specific markers, we considered anxiety, perfectionism, and athletic identity. To achieve this, our first step was to translate and validate a specific measure of irrational beliefs, namely the Irrational Performance Beliefs Inventory (iPBI). The iPBI is a performance-relevant measure that captures specific IB, taking into account the situational circumstances of the target population, namely operators in different performance context (academia, sports, business, medicine, etc.). Its theoretical basis is largely Ellis’ work on rational and irrational beliefs. We developed a short and a long version of the iPBI, which both capture four core IBs (i.e., demandingness, awfulization, low frustration tolerance, and depreciation). Factorial validity was confirmed by a confirmatory factor analysis (comparative fit index = 0.92) with data from 234 athletes. Both versions of the newly developed iPBI showed good internal consistency (Cronbach’s alpha > 0.77) and retest reliability (intra-class correlation coefficients >0.71). Results of the correlational analyses indicated low positive relationships between IBs and athletes’ psychological distress, and low negative relations between IBs and wellbeing. In terms of sport-specific markers, there were low to moderate correlations with IBs. This study also examined the differences in IBs between females and males, individual and team sport athletes, and across three different performance levels. Implications of these findings are discussed along with approaches for future research and applied work.

## Introduction

Performance is a part of everyday life for most people. At work, at school, or in sports, there may be repeated experience with failure, unfairness, rejection, or similar challenging events to which a person may respond with healthy or unhealthy behaviors (Chotpitayasunondh and Turner, 2019). In this regard, healthy behavioral responses can be described as adaptive, having a motivating and goal promoting effect and based on rational beliefs that are logical and/or pragmatic with an empirical basis (Szentagotai and Jones, 2010). In contrast, unhealthy behaviors can be demotivating, performance limiting and stressful (Szentagotai and Jones, 2010) and are based on irrational beliefs (IBs), which are defined as illogical, unpragmatic, and empirically unsubstantiated. Moreover, compared to rational beliefs, IBs lead to an inflexible lack of acceptance for non-preferred situational outcomes, that is, individuals may over-invest in a particular desirable outcome while having an unwillingness to accept an undesirable alternative outcome (Davies, 2008; Dryden, 2009; David et al., 2010). IBs are at the heart of treatment in Rational Emotive Behavior Therapy (REBT) and are therefore an essential component of REBT theory and practice. According to this theory (REBT; Dryden, 2014), emotions and behaviors are not determined by an experienced situation alone, but by the cognitive processing of that situation (i.e., cognitive mediation; Turner et al., 2021). Thus, irrational compared to rational beliefs about an event can lead to emotional responses that are dysfunctional in terms of goal attainment and physical and psychological wellbeing (Szentagotai and Jones, 2010; Turner and Barker, 2014). Indeed, extant literature indicates that irrational beliefs are associated with maladaptive affective responses (Vîslă et al., 2016) as well as dysfunctional behaviors such as self-harming or social isolation (Szentagotai and Jones, 2010). In summary, irrational beliefs can lead to inappropriate emotional and behavioral responses that subjectively burden individuals and prevent them from achieving their personal goals in life (Wilken, 2018). REBT, through its ABCDE framework (see Ellis, 1994), provides a specific intervention in which deeply held irrational beliefs are first assessed (recognizing that they cause undesirable emotional and behavioral consequences rather than the event itself), then rigorously disputed, and finally replaced with rational beliefs to improve individuals’ mental health. Accordingly, uncovering irrational beliefs can be essential by having a positive impact on individuals’ wellbeing and daily performance.

In the competitive sports context, identifying irrational beliefs and applying REBT are also playing an increasingly important role. In a recent systematic review of the area, Jordana et al. (2020) indicate that a reduction in IBs through REBT leads to decreased performance anxiety (e.g., Turner and Barker, 2013), increased self-acceptance (Cunningham and Turner, 2016), increased resilience (Deen et al., 2017), increased self-efficacy (e.g., Chrysidis et al., 2020), increased self-determined motivation (e.g., Davis and Turner, 2020), and increased performance (e.g., Wood et al., 2017). In addition, in athlete samples, greater endorsement of IBs is related to greater anxiety and depression (Chotpitayasunondh and Turner, 2019; Turner et al., 2019a,b, 2022; Mansell, 2021), greater trait anger (Turner et al., 2019a), greater performance anxiety (Chadha et al., 2019) and increased burnout (Turner and Moore, 2016), and worse performance under pressure (Turner et al., 2019c; Mesagno et al., 2021).

In order to identify IBs in athletes, to weaken IBs and the associated burdens in the future, and to evaluate the effectiveness of REBT interventions, an instrument that measures the extent of IBs is needed. Thus, Turner et al. (2018a) developed the Irrational Performance Beliefs Inventory (iPBI) from which an athlete version (iPBI-2) emerged (Turner and Allen, 2018). The aforementioned iPBI (Turner et al., 2018a) is a 28-item psychometric instrument that measures irrational performance beliefs specific to the performance domain (e.g., occupational, sports, military, and college). The iPBI reliably measures four core IBs according to REBT theory (Ellis, 1957; Dryden, 2014; Bernard and Dryden, 2019). Demandingness, also known as primary irrational belief (PIB), represent the first core belief and can be defined as rigid assertion of desires and demands that certain conditions must or must not exist (DiGiuseppe, 1996). Subordinate to the PIB are three further secondary IBs: Awfulizing (AWF) defined as the overestimation of the consequences of past, present, and future events while being unable to recognize that there could be worse events (David et al., 2010). Low-frustration tolerance (LFT) which describes the belief that when people do not get what they think they should get, they conclude that the situation is unbearable, and they cannot stand it (Dryden, 2002). Lastly, depreciation (DEP) that refers to the natural tendency to make global evaluations (i.e., overgeneralize) about themselves, others, and the world (David et al., 2010). These beliefs are considered the four core beliefs out of the original 11 (Ellis, 1962) and are related to performance and performance-related aspects (e.g., achievement, approval, and failure) in the iPBI.

In addition to achieving optimal athletic performance, athletes face many demands that can affect their emotional, cognitive, and behavioral wellbeing. For example, an athlete’s success or failure in a particular competition can have far-reaching consequences on financial support, sponsoring-contracts, qualifications for higher-level competitions, or nomination to a squad. This may lead athletes to attach irrational personal importance to an event (Ellis, 2002), which may also trigger athletes’ PIBs, for example, “I have to win” or “I have to be the best” in order to achieve perfection and thus a specific goal such as being nominated to a team. The dysfunctionality behind this lies in the fact that only absolute perfectionism is considered a success, and failure to achieve it can affect the athlete’s self-worth and wellbeing (Flett et al., 2003). Moreover, by striving for perfectionistic performance and achieving specific athletic goals, athletes may also run the risk of over-identifying with their role as an athlete at the expense of other areas of life (e.g., job, family, friends, and other social commitments; Heird and Steinfeldt, 2013). Driven by their IBs, athletes can develop a range of negative emotions (e.g., anxiety and guilt), when they miss a training session, or they may develop dysfunctional training behaviors, such as training during an injury, overtraining, or obsessive training behaviors that affect their physical and mental health (Heird and Steinfeldt, 2013; Outar et al., 2018). Therefore, one of our aims was to consider perfectionism and athletic identity as relevant markers of athletes’ mental health and to identify possible associations with IBs in order to support athletes in healthy performance development through specific interventions in the future.

In the interest of measuring athlete IBs cross-culturally, several translations of the iPBI have been developed and tested for validity: Persian (Nejati et al., 2021), Thai (Chotpitayasunondh and Turner, 2019), and Turkish (Urfa and Aşçı, 2018). However, to date, an assessment of IBs in German athletes has not been achieved, in part because no measure of IBs in German language has been developed and robustly tested. Thus, at present, it is not known to what extent the IBs of German athletes are a risk factor for mental health issues such as anxiety and depression. In the German-speaking area, there are so far only a few measures for assessing IBs (e.g., Questionnaire of Irrational Attitudes, Klages, 1989; Dysfunctional Attitude Scale, Hautzinger et al., 2005). However, these are mainly applied in a clinical context, are not context-specific, and do not take into account the situational circumstances of the target population of athletes. As such, it is not known whether and to what extent IBs are prevalent in German athletes or how these IBs might be associated with important markers of athlete mental health (i.e., anxiety, depression, wellbeing, perfectionism, and athletic identity). Therefore, the purpose of the current paper was to investigate these relationships. To achieve this examination, a rigorous and detailed translation, cultural adaptation, and validation process was undertaken (e.g., Wild et al., 2005) in order to develop a German version of the iPBI (G-iPBI). Only with a valid and reliable measure in German language could we accurately assess the IBs of German athletes.

## Materials and Methods

### Participants

The study comprised 234 athletes (124 woman and 110 men; age: *M* = 27.48, *SD* = 12.16) from 47 disciplines of various team- and individual sports. Athletes from soccer (*n* = 53), swimming as well as lifesaving sports (*n* = 27), handball (*n* = 22), athletics (*n* = 18), field hockey (*n* = 14), and triathlon (*n* = 13) were the most represented. The other 87 athletes were distributed among other sports, such as volleyball, basketball, golf, and many others. On average, athletes reported exercising 9.58 (*SD* = 4.86) hours per week. The sample included 102 amateur athletes, 100 semi-professional athletes, and 32 competitive athletes. Amateur athletes were defined as those performing in local competitions and who were not receiving remuneration as part of their sport participation (except for soccer players who may also earn money in the fifth league or below). Semi-professional athletes were defined as those performing at national and international level and receiving some remuneration as part of their sport participation depending on the discipline and competitive-elite athletes regularly compete at the highest level in their sport.

### Measures

For all questionnaires, we used the German versions. Descriptive statistics as well as reliability coefficients (Cronbach’s alpha; α) for the present sample are displayed on the left side of Table 1.

**Table 1:** Means (M), standard deviations (SD), and Cronbach’s alphas (α) of the construct of concurrent validity, corresponding mental health markers, and social desirability as well as respective correlations with G-iPBI dimensions.

	*M* (*SD*)	Min	Max	*α*	PIB		LFT		AWF		DEP		COMP	
					26	20	26	20	26	20	26	20	26	20
**Concurrent validity**														
**DAS-A-17**														
Perfectionism	25.10 (11.36)	11	77	0.91	0.37^***^	0.37^***^	0.35^***^	0.33^***^	0.40^***^	0.39^***^	0.69^***^	0.70^***^	0.57^***^	0.57^***^
Dependency	20.54 (7.40)	6	42	0.85	0.54^***^	0.54^***^	0.34^***^	0.30^***^	0.54^***^	0.54^***^	0.57^***^	0.60^***^	0.60^***^	0.61^***^
**General markers**														
**WHO-5**	14.18 (4.47)	0	25	0.78	−0.25^**^	−0.25^**^	−0.21^*^	.19 *ns*.	−0.25^**^	−0.22^*^	−0.39^***^	−0.39^***^	−0.34^***^	−0.33^***^
**GAD-7**	19.07 (3.67)	0	21	0.81	0.30^***^	0.30^***^	0.27^***^	0.26^**^	0.35^***^	0.32^***^	0.53^***^	0.54^***^	0.46^***^	0.45^***^
**PHQ-9**	23.87 (4.39)	0	27	0.82	0.30^***^	0.30^***^	0.31^***^	0.31^***^	0.34^***^	0.32^***^	0.51^***^	0.51^***^	0.46^***^	0.45^***^
**Sports-specific markers**														
**WAI-T**														
Somatic Anxiety	9.65 (3.06)	4	16	0.84	0.20^*^	0.20^*^	0.11 *ns*.	0.11 *ns*.	0.23^**^	0.22^*^	0.39^***^	0.37^***^	0.29^***^	0.29^***^
Worry	9.33 (3.34)	4	16	0.89	0.32^***^	0.32^***^	0.28^***^	0.28^***^	0.37^***^	0.36^***^	0.54^***^	0.55^***^	0.48^***^	0.48^***^
Concentration d.	6.44 (2.21)	4	16	0.67	0.25^**^	0.25^**^	0.13 *ns*.	0.10 *ns*.	0.24^**^	0.20^*^	0.31^***^	0.30^***^	0.28^***^	0.27^**^
**MIPS-Training**														
Demands	29.04 (9.40)	8	48	0.94	0.20 *ns*.	0.20^*^	0.34^***^	34^***^	0.26^**^	0.28^***^	0.13 *ns*.	0.15 *ns*.	0.28^***^	0.29^***^
Negative reactions	20.71 (7.84)	8	48	0.92	0.34^***^	0.34^***^	0.42^***^	0.42^***^	0.41^***^	0.41^***^	0.49^***^	0.51^***^	0.51^***^	0.53^***^
**MIPS-Competition**														
Demands	25.47 (8.42)	8	48	0.91	0.45^***^	0.45^***^	0.46^***^	0.46^***^	0.50^***^	0.49^***^	0.56^***^	0.58^***^	0.60^***^	0.61^***^
Negative reactions	31.70 (10.18)	8	48	0.94	0.33^***^	0.33^***^	0.43^***^	0.44^***^	0.36^***^	0.37^***^	0.19 *ns*.	0.21^*^	0.39^***^	0.41^***^
**Athletic identity**	36.82 (7.56)	7	49	0.84	0.23^*^	0.23^*^	0.27^**^	0.29^***^	0.25^*^	0.27^**^	0.06 *ns*.	0.05 *ns*.	0.23^*^	0.25^*^
Social identity	16.57 (3.26)	3	21	0.69	0.09 *ns*.	0.11 *ns*.	0.07 *ns*.	0.14 *ns*.	0.08 *ns*.	0.13 *ns*.	0.22 *ns*.	−0.09 *ns*.	0.33 *ns*.	0.08*ns*.
Exclusivity	9.51 (2.85)	2	14	0.80	0.16 *ns*.	0.16 *ns*.	0.15 *ns*.	0.16 *ns*.	0.16 *ns*.	0.18 *ns*.	0.06 *ns*.	0.04 *ns*.	0.15 *ns*.	0.16*ns*.
Negative affectivity	10.74 (2.85)	2	14	0.71	0.32^***^	0.32^***^	0.42^***^	0.44^***^	0.36^***^	0.38^***^	0.19 *ns*.	0.20 *ns*.	0.38^***^	0.40^***^
**Social desirability**	2.19 (1.00)	0	4	0.26	−0.19^**^	−0.19^**^	−0.12^*^	0.12 *ns*.	−0.20^**^	−0.18^*^	−0.25^***^	−0.26^***^	−0.24^**^	−0.24^**^

ns., not significant; Min/Max, potential lowest/highest value on the respective (sub)scale; 26, 26-item version of the German Irrational Performance Beliefs Inventory; 20, 20-item version of the German Irrational Performance Beliefs Inventory; PIB, Primary irrational beliefs; LFT, low frustration tolerance; AWF, awfulization; DEP, depreciation; COMP, Composite score of the respective G-iPBI version; DAS-A-17, Dysfunctional Attitude Scale Form A revised (De Graaf et al., 2009); WHO-5, Well-being index (Brähler et al., 2007); GAD-7, General Anxiety Disorder Questionnaire (Löwe et al., 2008); PHQ-9, Patient Health Questionnaire (Gräfe et al., 2004); WAI-T, Competition Anxiety Inventory (Brand et al., 2009); Concentration *d.*, Subscale Concentration difficulties of the WAI-T; MIPS, Multidimensional Inventory of Perfectionism in Sports (Stöber et al., 2004); Demands, Subscale Self-related perfectionist demands of the MIPS; Negative reactions, Subscale Self-related negative reactions to non-perfect performance of the MIPS; Composite score 26-item G-iPBI: *M* = 81.27, *SD* = 16.69, *α* = 0.94; and Composite score 20-item G-iPBI: *M* = 63.51, *SD* = 12.69, *α* = 0.92.

* *p* < 0.05;

** *p* < 0.01;

*** *p* < 0.001.

#### Irrational Beliefs

In its original version, the iPBI (Turner et al., 2018a) consists of 28 items, with seven items assigned to each dimension of irrational beliefs: PIB (e.g., “I must not be dismissed by my peers.”), LFT (e.g., “I cannot bear not getting better at what I do”), AWF (e.g., “It’s terrible if the members of my team do not respect me”), and DEP (e.g., “I am a loser if I do not succeed in things that matter to me”). However, one item could not be easily related equally to all performance contexts (i.e., “I need my coach to act respectfully toward me”). Thus, Turner and Allen (2018) developed a second, shorter version of the iPBI (iPBI-2) with only 20 items to continue to ensure validity in different performance contexts (Turner and Allen, 2018). In the iPBI-2, the dimensions each consist of five items from the original long version. In both versions, athletes rated the different statements on a five-point scale from “1” (strongly disagree) to “5” (strongly agree). Cronbach’s α for the original study lies 0.90 and 0.96 (Turner et al., 2018a) for the iPBI and between 0.76 and 0.87 (Turner and Allen, 2018) for the iPBI-2.

For the present study, we translated the original English version of the iPBI into German following Wild et al. (2005) (for a similar approach, see Chotpitayasunondh and Turner, 2019). We considered the following steps in our translation process: First, two independent translators from the field of sports psychology carried out the forward translation of the measure. Second, the two forward translations were merged into a single forward translation, always in consultation with the author of the original scale to best match the intensity and meaning of individual words. In step 3, five independent translators who were either native speakers or fluent in English performed the back translation of the measure. In step 4, the harmonization phase, all back-translated versions were compared with each other and with the original version. This was initially done together with the back translators. The result of step 4 was a preliminary final version. Last, we identified partial changes to the translation that are necessary for improvement. Therefore, the items have been reworded where necessary. This resulted in the final translation of the measure, which was used after a final proofreading.

As a second measure of irrational beliefs, we used the Dysfunctional Attitude Scale Form A revised (DAS-A-17; De Graaf et al., 2009), which determines the intensity of dysfunctional attitudes with two subscales. Subscale 1 Perfectionism/Performance evaluation consists of 11 items (e.g., “If a person asks for help, it is a sign of weakness”) and contains items about perfectionism and concerns about being negatively evaluated by others based on their performance. Subscale 2 Dependency includes six items (e.g., “If others dislike you, you cannot be happy”) and captures the need to lean on, being supported by others, as well as the dependency of approval and judgments by others in the context of interpersonal relations. Items were rated on a seven-point Likert scale from “7” (fully agree) to “1” (fully disagree). Cronbach’s α for the original study is 0.90 for Perfectionism/Performance evaluation subscale, 0.81 for Dependency subscale, and 0.91 for the DAS-A-17 total score.

#### Psychological Distress and Well-Being

##### Psychological Distress

We used two established measures—the Patient Health Questionnaire (PHQ-9; Gräfe et al., 2004, English original by Spitzer et al., 1999), and the General Anxiety Disorder Questionnaire (GAD-7; Löwe et al., 2008, English original by Spitzer et al., 2006) to assess psychological distress. The PHQ-9 consists of nine statements (e.g., “Trouble falling or staying asleep, or sleeping too much”) that assess frequency in symptoms of depression and the GAD-7 consists of seven items (e.g., “Feeling afraid as if something awful might happen”) that measure anxiety symptoms. Both scales refer to the period of the last two weeks and were rated on a four-point scale from “0” (not at all) to “3” (nearly every day). Higher values indicate a higher degree of symptomatology. Cronbach’s *α* is 0.88 (PHQ-9; Gräfe et al., 2004) and 0.89 (GAD-7; Löwe et al., 2008) for the original studies.

##### Well-Being

We applied the WHO-5 Well-being index (WHO-5, Brähler et al., 2007) to evaluate participants’ wellbeing at the time of the survey. The WHO-5 includes five statements (e.g., “…I was happy and in a good mood”) to be answered for the period of the last two weeks. Response format is a six-point scale ranging from “0” (at no time) to “5” (all the time). Unlike the psychological distress test scores, higher scores on the WHO-5 are interpreted with lower wellbeing. Cronbach’s α is 0.92 for the original study (Brähler et al., 2007).

#### Social Desirability

We used the short version of the Social Desirability Scale (SDS-CM; Lück and Timaeus, 2014) to assess error influence in our survey, i.e., responses that were voted on believing that they met the approval of others. The SDS-CM consists of four statements, which participants answered with either a “yes” or a “no.” A score of “1” was allocated to answers that deemed to be the socially desirable answer. Cronbach’s α is 0.60 for the original study (Lück and Timaeus, 2014).

#### Sport Specific Measures

##### Sport-Specific Competition Anxiety

With the Competition Anxiety Inventory (WAI-T; Brand et al., 2009; see also Sport Anxiety Scale, Smith et al., 1990) we assessed athletes’ sport-specific competition anxiety. The WAI-T consists of three subscales with four items each: Worry (e.g., “I am concerned about choking under pressure.”), Somatic anxiety (e.g., “I feel nervous.”), and Concentration difficulties (e.g.,” I pay attention to reactions of spectators”). Athletes rated on a four-point scale from “1” (not at all) to “4” (very) how well the items describe their general thoughts and feelings before a competition. Cronbach’s α is 0.81 (Somatic anxiety), 0.83 (Worry), and, 0.77 (Concentration difficulties; Brand et al., 2009) for the original study.

##### Sports-Specific Perfectionism

The Multidimensional Inventory of Perfectionism in Sports (MIPS; Stöber et al., 2004) captures perfectionism in sports on multiple levels. Here we used two subscales, namely Self-related perfectionist demands and Self-related negative reactions to non-perfect performance. Both subscales can be divided into a training and a competition context, each of which is assessed with eight items. Item examples are “In training I want to do everything perfectly,” and “In the competition/league game, I want to do everything perfectly.” for self-related perfectionist demands. “In training, I get frustrated when I do not meet my extremely high expectations,” and “I get frustrated in competitions/league games when I do not meet my extremely high expectations.” are item exampled for self-related negative reactions. Cronbach’s α is between 0.92 and 0.95 (Self-related perfectionist demands) and between 0.86 and 0.91 (Self-related negative reactions to non-perfect performance) for the original study (Stöber et al., 2004).

##### Athletic Identity

We measured athletic identity using the Athletic Identity Measurement Scale (AIMS-D; Schmid and Seiler, 2003; English original by Brewer et al., 1993), which consists of seven items and is answered on a seven-point scale ranging from “1” (does not apply at all) to “7” (fully applies). In addition to the calculation of a total test score, the scale also allows the calculation of three subscales: Social identity (three items, e.g., “I consider myself an athlete”), Exclusivity (two items, e.g., “Sports are the most important part of my life”), and Negative Affectivity (two items, e.g., “I feel bad about myself when I do poorly in sport”). Cronbach’s α is 0.54 (Social Identity), 0.74 (Exclusivity), 0.62 (Negative Affectivity), and 0.74 (total test score) for the original study (Schmid and Seiler, 2003).

### Procedure

Athletes from various team and individual sports throughout Germany were invited to participate in the study by email *via* their respective clubs or sports associations. The study was conducted online *via* SoSci-Survey (Leiner, 2019), and in compliance with the Declaration of Helsinki (World Medical Association, 2013). Ethical approval was granted from the local Review Board of the Institute for Psychology of the Johannes Gutenberg-University. Participants were informed about the nature and the procedure of the study. They gave their informed consent before completing the questionnaires. Participation was voluntary and participants received no incentives. Participation requirements were a minimum age of 16 years and the exercise of a sporting activity.

### Data Analyses

We performed statistical analyses using R Studio (R Core Team, 2018).

#### Data Screening

First, we checked our data for univariate and multivariate normal distribution using Shapiro–Wilk-test for univariate normality and Mardia’s coefficient for multivariate normality (“mvn”-package; Korkmaz et al., 2014). The analyses revealed neither a normal distribution for the individual items nor a multivariate normal distribution of the data (Mardia Kurtosis 16.26, *p* < 0.001; Mardia, 1970; Tabachnick and Fidell, 2013). Thus, we considered the data suitable for confirmatory factor analysis (CFA) using robust maximum likelihood estimation (MLR), that computes standard errors and model fit indices that are robust in relation to the relative non-normality of observations (Hu et al., 1992). Second, we screened data for outliers (standardized *z* values >3.29; Tabachnick and Fidell, 2013, Mair and Wilcox, 2020), and winsorized outliers at time 1 (*n* = 15 from 6,552 cases <0.01%), and at time 2 (*n* = 4 from 2,380 cases <0.01%).

#### Confirmatory Factor Analysis

The translated 28 IPBI-items were subjected to statistical validation analysis, for which a sample size of five participants per item is recommended (DeVellis, 2016). For this purpose, a CFA was first conducted to test the four-factor structure of the iPBI in the German sample of athletes. We assessed the goodness of model fit with multiple fit indices and reported the χ^2^-test statistic, the Root Mean Square Error of Approximation (RMSEA) and its confidence interval (90% CI), as well as the Standardized Root Mean Square Residual (SRMR), the Comparative Fit Index (CFI), and the Tucker Lewis Index (TLI). RMSEA-values less than 0.08 indicate an acceptable model and less than 0.06 indicate a good model (Hu and Bentler, 1999). For the SRMR index, values should be <0.05 for a good fit and <0.10 for an acceptable fit. Regarding CFI und TLI index, values close to 0.95 are indicative of a good model fit (Hu and Bentler, 1999).

#### Correlational Analyses

To test the criterion validity of our measure, we first correlated the respective G-iPBI subscales with an existing measure of dysfunctional attitudes (DAS-A-17; concurrent validity). Because the DAS-A-17 covers only a subset of IB dimensions, we expected moderate to high correlations between these two measures. Second, we investigated the relationship between IBs and important markers of mental health (PHQ-9, GAD-7, and WHO-5). Based on the results of previous studies (Turner et al., 2018a, 2019a), we also expected a low correlation between the four dimensions of IBs with athletes’ psychological distress and wellbeing. To explore the relations between IBs and sport-specific markers, we additionally correlated G-iPBI and G-iPBI-2 with measures of sport-specific anxiety (WAI-T), sport-specific perfectionism (MIPS), and athletic identity (AIMS-D). In accordance with Hinkle et al. (2003), correlation coefficients are rated as low (0.30–0.50), moderate (0.50–0.70), high (0.70–0.90), and very high (0.90–1.00).

#### Reliability Analyses

We computed internal reliability coefficients (Cronbach’s *α*) for each IB subscale. According to Nunnally and Bernstein (1994), coefficients greater than 0.70 indicate good test score reliability and coefficients greater than 0.90 indicate excellent test score reliability. In addition, we examined the test–retest reliability over a two-week interval to test the stability and the reproducibility of our measures and calculated intra-class correlation coefficients (ICC). According to Koo and Li (2016), values greater than 0.90 are excellent, values between 0.75 and 0.90 are good, values between 0.50 and 0.75 are moderate, and values less than 0.50 describe a poor reliability.

#### Population-Based Differences

First, we correlated subscales of the both G-iPBI versions with participants’ age. Referring to the results of previous studies (Turner and Allen, 2018; Turner et al., 2018a), we expected that younger athletes report higher levels of irrational beliefs than older athletes. In addition, we divided our sample into three different performance levels (amateur vs. semi-elite vs. competitive-elite) following Swann et al. (2015) and two different sport-types (team vs. individual athletes). Moreover, building on previous findings (Turner and Allen, 2018, Turner et al., 2018a) indicating gender differences in irrational beliefs, we also examined differences between men and women. To this end, in a second step, we conducted a three-way MANOVA with performance level, sport-type, and gender to examine differences in the four dimensions of irrational beliefs. Beforehand, we tested the assumption of independent observations with intra-correlations of the dependent variables as well as multivariate normality with the Shapiro–Wilk-test (*p* > 0.05) and equality of variance–covariance matrices with Box’s M test (*p* > 0.05). In the case of a violation of the assumptions, we then performed robust ANOVAs with trimmed means (20%) using the t3way function from WRS2 package (Mair and Wilcox, 2020) to determine mean differences in the different groups after obtaining significant main effects. In addition, we reported partial eta squared (*η_p_*^2^) as corresponding effect size with the following criteria for small, medium, and large effect: 0.01, 0.06, and >0.14 (Vacha-Haase and Thompson, 2004; Fritz et al., 2012). Based on previous findings (Turner and Allen, 2018, Turner et al., 2018a), we hypothesized that women report higher levels of irrational beliefs than men. Because results were mixed in the previous studies (Turner and Allen, 2018; Nejati et al., 2021), we did not make any specific hypotheses regarding performance level and sport-type.

However, given the data we collected to test the validity of our measure, we were also able to conduct some exploratory analyses of other differences among athletes’ gender, performance level, and sport-type in psychological distress, wellbeing, as well as competitive anxiety and perfectionism to provide further information for researchers, coaches, and practitioners. The results of the exploratory analyses are summarized in the Supplementary Material.

## Results

### Construct Validity

We performed a CFA to examine the factorial validity of the G-iPBI and evaluated factor loadings, error variances, and modification indices. Due to the non-normal distribution of our data, we applied robust maximum likelihood estimation with Yuan-Bentler scaled test statistic (Hu et al., 1992). First, we tested whether the original four-factor structure of 28-item iPBI could be confirmed in our German sample. Results revealed a somewhat unacceptable fit to the expected 28-item model, *N* = 234, *χ*^2^(344) = 840.81, *p* < 0.001, CFI = 0.856, TLI = 0.842, SRMR = 0.072, RMSEA = 0.081 (90% CI: 0.074, 0.088). In particular, item 1 (“Decisions that affect me must be justified”) and item 7 (“I need my manager/coach to act respectfully towards me”) from the PIB dimension proved to be problematic due to low factor loadings (<0.50; Costello and Osborne, 2005). Therefore, we carried out a second CFA in which these two items were excluded. In addition, we allowed the residuals of item 22 and 25 of the DEP dimension, item 15 and 21 of the AWF dimension, and item 13 and 14 of the LFT dimension to co-vary. Results indicated an acceptable fit to the 26-item model: *N* = 234, *χ*^2^(290) = 568.48, *p* < 0.001, CFI = 0.916, TLI = 0.906, SRMR = 0.068, RMSEA = 0.066 (90% CI, 0.058, 0.074). Table 2 shows the final standardized solution and fit statistics for the four-factor 26-item G-iPBI. Factor loadings ranged from 0.56 to 0.89, and error variances were between 0.20 and 0.69.

**Table 2:** Standardized solution and fit statistics for the four-factor 26-item and 20-item German version of the iPBI (G-iPBI; values in parentheses are for the 20-item G-iPBI).

Nr.	Items				Error variances	Standardized factor loadings			
						PIB	LFT	AWF	DEP
2	I have to be viewed favourably by people that matter to me. *Ich muss von Menschen, die mir wichtig sind, positiv gesehen werden.*				0.69 (0.66)	0.56 (0.58)			
3	I need others to think that I make a valuable contribution. *Ich brauche es, dass Andere denken, dass ich einen wertvollen Beitrag leiste.*				0.59 (0.58)	0.64 (0.64)			
4	I absolutely should not be snubbed by people that matter to me. *Ich sollte absolut nicht von Menschen, die mir wichtig sind, abgewiesen werden.*				0.53 (0.53)	0.68 (0.69)			
5	I must not be dismissed by my peers. *Ich darf von meinen Teamkollegen nicht abgelehnt werden.*				0.48 (0.49)	0.72 (0.72)			
6	I have to be respected by the members of my team. *Ich muss von Mitgliedern meines Teams respektiert werden.*				0.62 (0.64)	0.62 (0.60)			
8	I cannot bear not being given chances. *Ich kann es nicht ertragen, keine Chancen zu bekommen.*				0.58		0.65		
9	I cannot stand not reaching my goals. *Ich kann es nicht ertragen, meine Ziele nicht zu erreichen.*				0.61 (0.65)		0.62 (0.59)		
10	I cannot bear not succeeding in things that are important to me. *Ich kann es nicht ertragen, in Dingen, die mir wichtig sind, nicht erfolgreich zu sein.*				0.36 (0.37)		0.80 (0.79)		
11	I cannot tolerate it when I fail at something that means a great deal to me. *Ich kann es nicht ertragen, wenn ich bei etwas scheitere, das mir sehr viel bedeutet.*				0.45		0.74		
12	I cannot stand failing in things that are important to me. *Ich kann es nicht ertragen, in Dingen zu versagen, die mir wichtig sind.*				0.37 (0.38)		0.80 (0.79)		
13	I cannot bear not getting better at what I do. *Ich kann es nicht ertragen, nicht besser zu werden in dem, was ich tue.*				0.56 (0.48)		0.66 (0.72)		
14	I could not stand it if my competencies did not continually develop and improve. *Ich könnte es nicht ertragen, wenn sich meine Kompetenzen nicht ständig weiterentwickeln und verbessern.*				0.60 (0.52)		0.63 (0.69)		
15	It’s awful to not be treated fairly by my peers. *Es ist schrecklich, von meinen Teamkollegen nicht fair behandelt zu werden.*				0.61			0.62	
16	It’s awful if others do not approve of me. *Es ist schrecklich, wenn andere mich nicht anerkennen.*				0.43			0.75	
17	It’s awful if others think I do not make a valuable contribution. *Es ist schrecklich, wenn andere denken, dass ich keinen wertvollen Beitrag leiste.*				0.50 (0.50)			0.70 (0.71)	
18	It would be terrible to be dismissed by my peers. *Es wäre schrecklich, von meinen Teamkollegen abgelehnt zu werden.*				0.47 (0.50)			0.73 (0.70)	
19	It is appalling if others do not give me chances. *Es ist entsetzlich, wenn andere mir keine Chancen geben.*				0.69 (0.70)			0.56 (0.54)	
20	It would be awful if my position in my team was not secure. *Es wäre schrecklich, wenn meine Position in meinem Team nicht sicher wäre.*				0.59 (0.58)			0.64 (0.65)	
21	It’s terrible if the members of my team do not respect me. *Es ist schrecklich, wenn die Mitglieder meines Teams mich nicht respektieren.*				0.54 (0.62)			0.67 (0.66)	
22	If decisions that affect me are not justified, it shows that I am worthless. *Wenn Entscheidungen, die mich betreffen, nicht gerechtfertigt sind, zeigt das, dass ich wertlos bin.*				0.45				0.74
23	If others think I am no good at what I do, it shows I am worthless. *Wenn andere denken, dass ich nicht gut in dem bin, was ich tue, zeigt das, dass ich wertlos bin.*				0.20 (0.20)				0.89 (0.89)
24	If I face setbacks it goes to show how stupid I am. *Wenn ich Rückschläge erleide, zeigt das, wie dumm ich bin.*				0.39 (0.39)				0.78 (0.78)
25	If I am not given opportunities, then it shows that I am not a worthwhile person. *Wenn mir keine Chancen gegeben werden, dann zeigt es, dass ich keine wertvolle Person bin.*				0.43				0.76
26	I am a loser if I do not succeed in things that matter to me. *Ich bin ein Versager, wenn ich in Dingen, die mir wichtig sind, nicht erfolgreich bin.*				0.34 (0.35)				0.81 (0.81)
27	If my position in my team was not secure, then it would show I am worthless. *Wenn meine Position in meinem Team nicht sicher wäre, dann würde das zeigen, dass ich wertlos bin.*				0.29 (0.30)				0.84 (0.84)
28	If my competencies did not continually develop and improve, it would show what a failure I am. *Wenn sich meine Kompetenzen nicht ständig weiterentwickeln und verbessern, würde das zeigen, was für ein Versager ich bin.*				0.33 (0.31)				0.82 (0.83)
	Factor	Mean	*SD*	Skew	*α*	Inter-factor correlations			
	Primary irrational beliefs (PIB)	18.33 (18.33)	3.42 (3.42)	−0.81 (−0.81)	0.77 (0.77)	-			
	Low frustration tolerance (LFT)	24.59 (17.75)	5.27 (3.71)	−0.55 (−0.54)	0.87 (0.84)	0.54 (0.50)	-		
	Awfulizing (AWF)	24.22 (17.18)	4.97 (3.67)	−0.48 (−0.43)	0.85 (0.78)	0.78 (0.77)	0.65 (0.61)	-	
	Depreciation (DEP)	14.20 (10.29)	6.33 (4.62)	0.96 (0.88)	0.93 (0.92)	0.50 (0.48)	0.49 (0.47)	0.56 (0.53)	-

Numbering of the items refers to the original version (Turner et al., 2018a) for better comparability. All inter-factor correlations are significant at the 0.001 level. *p* values were adjusted for multiple testing (Holm’s method). Composite score 26-item G-iPBI: *M* = 81.27, *SD* = 16.69, *α* = 0.94; Composite score 20-item G-iPBI: *M* = 63.51, *SD* = 12.69, *α* = 0.92.

Second, we investigated whether the 20-item short version of the iPBI (iPBI-2; Turner and Allen, 2018) could also be mapped in our sample. The CFA produced an acceptable fit to the 20-item measure (G-iPBI-2) with the following model fit indices: *N* = 234, *χ*^2^(163) = 339.33, *p* < 0.001, CFI = 0.920, TLI = 0.904, SRMR = 0.062, RMSEA = 0.071 (90% CI: 0.060, 0.081). Again, based on the modification indices and due to the content affinity of the items, we allowed the residuals of item 6 (DEM dimension) and 21 (AWF dimension) to co-vary.^1^ Factor loadings (0.54–0.89) and error variances (0.20–0.69) were in a similar range as for the 26-item version (see Table 2).

### Concurrent Validity and Scale Reliability

We first tested concurrent validity through correlations between both versions of the G-iPBI, and the DAS-A-17 subscales Perfectionism/Performance evaluation and Dependency. The right part of Table 1 shows low to medium positive correlations between subscales of the G-iPBI and both subscales of the DAS-A-17. The highest correlation was between Perfectionism/Performance evaluation and DEP (*r*_26_ = 0.69; *r*_20_ = 0.70), followed by Dependency and the iPBI composite score (*r*_26_ = 0.60; *r*_20_ = 0.61), as well as DEP (*r*_26_ = 0.57; *r*_20_ = 0.61). Moreover, Pearson’s correlation coefficients revealed small negative relationships between the four G-iPBI dimensions of both versions and social desirability, indicating that both versions of the G-iPBI may be slightly susceptible to response bias.

Second, we calculated Cronbach’s alpha coefficients as measures of internal consistency of the G-iPBI and its subscales. Table 2 shows that all alpha coefficients were in a good to excellent range (*α* > 0.77, Nunnally and Bernstein, 1994). According to the results of Table 3, the values of the ICC of the subscales obtained within a two-week interval ranged from 0.745 (PIB) to 0.860 (DEP) in the 26-item version. Results for the 20-item version ranged from 0.710 (LFT) to 0.818 (DEP). Values of all subscales were in a moderate to good range (Koo and Li, 2016), which indicates the acceptability of temporal reliability or repeatability of subscales in the G-iPBI. Also, the ICC for the composite scores with 0.860 (Ci: 0.792, 0.907) for the 26-item version and with 0.850 (Ci: 0.779, 0.900) for the 20-item version confirmed the test–retest reliability of the G-iPBI.

**Table 3:** Means (M), standard deviations (SD), Cronbach’s alphas (*α*) and intra-class correlation coefficients (ICC) with their confidence interval (95% CI) for the respective subscales and the composite negative thoughts score.

Factor	*M*	*SD*	*α*	ICC [95%CI]
G-iPBI				
Primary irrational beliefs	17.84	3.26	0.78	0.745 [0.634, 0.827]
Low frustration tolerance	24.98	4.81	0.85	0.780 [0.680,0.851]
Awfulizing	24.12	5.10	0.88	0.845 [0.770, 0.896]
Depreciation	14.19	6.38	0.95	0.823 [0.740, 0.881]
Composite Score	81.11	16.64	0.95	0.860 [0.793, 0.907]
G-iPBI-2				
Primary irrational beliefs	17.84	3.26	0.78	0.745 [0.634, 0.827]
Low frustration tolerance	17.81	3.53	0.83	0.710 [0.586, 0.801]
Awfulizing	16.95	3.77	0.82	0.848 [0.776, 0.899]
Depreciation	10.28	4.60	0.93	0.818 [0.733, 0.878]
Composite Score	62.88	12.66	0.93	0.851 [0.779, 0.900]

*N* = 85, G-iPBI, 26-item version of the German Irrational Performance Beliefs Inventory; G-iPBI-2, 20-item version of the German Irrational Performance Beliefs Inventory.

### Relationships Between IBs and Important Markers of Mental Health

We examined the relationships between G-iPBI as well as G-iPBI-2 and athletes’ general wellbeing and psychological distress (as measured by athletes’ anxiety and depression symptoms). Mainly low correlations emerged between G-iPBI subscales of both versions and psychological distress as well as athletes’ wellbeing. An exception was the correlation between psychological distress and DEP, which was moderate (see Table 1). In addition, we analyzed the correlations between the G-iPBI and G-iPBI-2 subscales and sports specific competition anxiety and perfectionism. The latter was separated in training and a competitive context. The respective G-iPBI subscales as well as the composite score of both versions showed low correlations with all three subscales of the WAI-T. Again, an exception was the correlation between DEP and the Worry subscale of the WAI-T, which was moderate (*r*_26_ = 0.54; *r*_20_ = 0.55). Regardless of context, low to moderate correlations emerged between the respective G-iPBI subscales and self-related perfectionistic demands, and self-related negative reactions. Interestingly, the correlations with the G-iPBI composite score and self-related negative responses were significantly higher than the correlations with the self-related perfectionistic demands related to training (composite scores: G-iPBI: *z* = 0.22, *p* < 0.001; G-iPBI-2: *z* = 3.13, *p* < 0.001). In the context of competition, the picture was reversed (composite scores: G-iPBI: z = 3.02, *p* < 0.001; G-iPBI-2: *z* = 2.94, *p* < 0.001). In addition, three of four IBs dimensions and athletic identity had significant but low correlations. However, this only applies to the subscale Negative affectivity and the overall athletic identity scale, while DEP is again the exception with non-significant correlations. The correlations with the subscales Exclusivity and Social identity were not significant.

### Population-Based Differences

Tables 4 provides Pearson correlation coefficients for athletes’ age as well as descriptive values (arithmetic mean values and standard deviations) for men and woman, amateur, semi-elite and competitive-elite athletes, as well as for individual and team athletes, for both the 26-item and the 20-item versions of the G-iPBI. Following Turner et al. (2019b), we conducted a three-way MANOVA with gender, performance level, and sport-type as independent variables to test whether potential effects might be nested across groups. In the following, we will first present the results of the 26-item and then the results of the 20-item version.

**Table 4:** Effect size differences between subgroups and bivariate correlations with participant age for the irrational beliefs’ dimensions.

	**Age**		**Gender**																			
	*r*		Men (*n* = 110)					Women (*n* = 124)													
			*M*	*SD*	*Md*	*IQR*	*M*	*SD*	*Md*	*IQR*											
**G-iPBI**																					
PIB	−0.27^***^		17.54	3.57	18	4	19.04	3.14	19	3											
LFT	−0.40^***^		23.73	5.77	24.5	8	25.35	4.67	26	5											
AWF	−0.27^***^		22.75	5.34	23	7.75	25.52	4.23	26	5											
DEP	−0.16^*^		12.34	5.34	11	6	15.85	6.69	15	9.25											
COMP	−0.32^***^		76.35	16.43	77	20	85.75	15.41	86.5	17											
**G-iPBI-2**																					
PIB	−0.27^***^		17.54	3.57	18	4	19.04	3.14	19	3											
LFT	−0.41^***^		17.12	4.14	18	6	18.31	3.2	19	4											
AWF	−0.28^***^		16.11	3.9	16	5	18.13	3.17	19	4											
DEP	−0.16^*^		8.96	3.94	8	5	11.47	4.87	11	6.5											
COMP	−0.33^***^		59.73	12.63	60	17	66.96	11.59	68	14											
**Performance level**													**Sport-type**							
Competitive-Elite (*n* = 32)				Semi-Elite (*n* = 100)				Amateur (*n* = 102)					Individual (*n* = 119)			Team (*n* = 115)				
	*M*	*SD*	*Md*	*IQR*	*M*	*SD*	*Md*	*IQR*	*M*	*SD*	*Md*	*IQR*	*M*	*SD*	*Md*	*IQR*	*M*	*SD*	*Md*	*IQR*
**G-iPBI**																				
PIB	18.22	3.17	18	4.25	18.09	3.61	19	4	18.61	3.32	19	3	17.86	3.71	18	4	18.82	3.05	19	3.5
LFT	25.69	3.95	27	5	23.81	5.95	24	8	25	4.84	26	5	24.33	5.22	25	7	24.85	5.33	26	5
AWF	24.81	4.55	25	5.5	23.64	5.04	24	7	24.59	5.01	26	6	23.76	5.19	25	8	24.69	4.7	26	6
DEP	13.66	5.62	12.5	8.25	14.52	6.76	13.5	10.25	14.05	6.14	14	8	15.07	7.01	14	10.5	13.29	5.42	13	7
COMP	82.37	12.38	83	15.25	80.06	18.2	83	25.25	82.25	16.05	83.5	18.75	81.02	18.11	83	26	81.65	14.84	84	17
**G-iPBI-2**																				
PIB	18.22	3.17	18	4.25	18.09	3.61	19	4	18.61	3.32	19	3	17.86	3.71	18	4	18.82	3.05	19	3.5
LFT	18.62	2.76	19	3.25	17.1	4.12	17.5	6	18.12	3.48	18.5	4	17.67	3.67	18	5	17.83	3.78	19	4
AWF	17.81	3.48	18	4.5	16.7	3.72	17	5	17.45	3.65	18	5	16.75	3.88	17	6	17.63	3.38	18	5
DEP	9.75	4.06	9	7	10.35	4.86	9	8	10.41	4.58	10	6	10.83	5.08	10	7	9.74	4.05	9	6
COMP	64.41	9.74	63.5	14.25	62.24	13.78	63	18	64.59	12.14	66	13	63.12	13.76	63	18	64.02	11.3	65	13.5

* *p* < 0.05;

*** *p* < 0.001.

#### G-iPBI (26 Items)

Results indicated that the four dimensions of IBs were not multivariate normally distributed (*W* = 0.980, *p* < 0.001). Moreover, the Box M results showed that the group variables performance level and sport-type had similar variance–covariance matrices (*p* > 0.05), in contrast to the gender variable (*p* < 0.05).

##### Age

The first analyses showed significant low negative correlations between athletes’ age and all subscales of the G-iPBI (*r* = −0.16 to −0.32), which implies that irrational beliefs decrease with age.

##### Gender

The three-way MANOVA indicated a significant main effect for gender, *λ* = 0.96, *F*(4,219) = 2.43, *p* = 0.049, *η_p_*^2^ = 0.042. A significant between-subject effect was revealed for all four IB dimensions: PIB, *F*(1,222) = 9.90, *p* < 0.01, *η_p_*^2^ = 0.041, LFT, *F*(1,222) = 5.38, *p* = 0.025, *η_p_*^2^ = 0.016, AWF, *F*(1,222) = 23.06, *p* < 0.001, *η_p_*^2^ = 0.079, and DEP, *F*(1,222) = 10.45, *p* < 0.01, *η_p_*^2^ = 0.049, indicating higher values on IB dimensions for woman compared to men (see upper part of Table 4).

##### Performance Level

There was no significant main effect for athletes’ performance level, *λ* = 0.95, *F*(8,438) = 1.52, *p* = 0.147, *η_p_*^2^ = 0.027.

##### Sport-Type

Further, results indicated a significant main effect for sport-type, *λ* = 0.95, *F*(4,219) = 2.84, *p* = 0.025, *η_p_*^2^ = 0.049. A significant between-subject effect was revealed for PIB, *F*(1,222) = 5.68, *p* = 0.024, *η_p_*^2^ = 0.021, with slightly higher values for team athletes (*M* = 18.82, *SD* = 3.05) than for individual athletes (*M* = 17.86, *SD* = 3.71) and for AWF, *F*(1,222) = 5.13, *p* = 0.027, *η_p_*^2^ = 0.01. Again, team athletes (*M* = 24.69, *SD* = 4.70) reported slightly higher AWF values compared to individual athletes (*M* = 23.76, *SD* = 5.19; see Table 4). There were no significant effects for LFT, *F*(1,222) = 0.21, *p* = 0.647, *η_p_*^2^ < 0.01, and DEP, *F*(1,222) = 2.10, *p* < 0.01, *η_p_*^2^ < 0.01.

Furthermore, there was a significant interaction effect for performance level and sport-type, *λ* = 0.90, *F*(8,438) = 2.43, *p* < 0.01, *η_p_*^2^ = 0.051. Significant interaction effects were revealed for AWF, *F*(2,222) = 14.34, *p* < 0.01, *η_p_*^2^ = 0.047, and LFT, *F*(2,222) = 10.91, *p* < 0.01, *η_p_*^2^ = 0.053, while no significant effects were obtained for PIB, *F*(2,222) = 2.85, *p* = 0.263, *η_p_*^2^ = 0.02, and DEP, *F*(2,222) = 2.65, *p* = 0.284, *η_p_*^2^ = 0.015. Subsequent *post-hoc* analyses revealed only a significant difference with respect to LFT (*p_bonf_* = 0.01) between semi-elite athletes in team sports (*M* = 22.32, *SD* = 7.00) and amateur athletes in team sports (*M* = 25.94, *SD* = 4.06). The upper part of Table 5 summarizes the means and standard deviations by performance level and sport-type of the athletes.

**Table 5:** Mean values (M), standard deviations (SD), median values (Md), and interquartile ranges (IQR) separated by performance level and sport-type of the athletes.

	Individual athletes				Team athletes			
	*M*	*SD*	*Md*	*IQR*	*M*	*SD*	*Md*	*IQR*
***G-iPBI***								
**PIB**								
Competitive-Elite	17.39	3.34	18.00	3.50	19.28	2.67	20.00	3.75
Semi-Elite	18.16	3.52	19.00	4.00	17.97	3.80	19.00	4.00
Amateur	17.60	4.19	19.00	5.75	19.20	2.54	19.50	3.00
**LFT**								
Competitive-Elite	25.00	4.69	25.50	6.25	26.57	2.62	27.00	1.75
Semi-Elite	24.68	5.09	26.00	7.00	22.32	7.00	23.00	11.00
Amateur	23.42	5.64	24.00	8.00	25.94	4.06	26.50	4.00
**AWF**								
Competitive-Elite	23.50	4.77	24.50	4.00	26.50	3.78	27.00	3.75
Semi-Elite	24.19	4.74	25.00	7.00	22.70	5.46	23.00	7.00
Amateur	23.16	6.09	24.00	9.50	25.45	4.05	26.00	5.00
**DEP**								
Competitive-Elite	13.72	5.93	12.00	7.75	13.57	5.42	12.50	8.75
Semi-Elite	15.94	7.15	14.00	9.50	12.11	5.31	11.00	6.00
Amateur	14.29	7.22	13.00	9.50	13.91	5.46	14.00	6.50
***G-iPBI-2***								
**PIB**								
Competitive-Elite	17.39	3.34	18.00	3.50	19.28	2.67	20.00	3.75
Semi-Elite	18.16	3.52	19.00	4.00	17.97	3.80	19.00	4.00
Amateur	17.60	4.19	19.00	5.75	19.20	2.54	19.50	3.00
**LFT**								
Competitive-Elite	18.33	3.27	18.50	5.50	19.00	1.96	20.00	1.75
Semi-Elite	17.73	3.64	19.00	5.00	16.03	4.69	16.00	8.00
Amateur	17.26	3.93	17.00	5.00	18.62	3.10	19.00	3.00
**AWF**								
Competitive-Elite	16.83	3.83	17.00	3.75	19.07	2.55	19.00	2.50
Semi-Elite	17.06	3.58	17.00	5.50	16.08	3.92	16.00	5.00
Amateur	16.18	4.40	17.00	7.50	18.20	2.90	18.50	4.00
**DEP**								
Competitive-Elite	9.55	4.17	8.50	6.25	10.00	4.06	9.50	7.00
Semi-Elite	11.28	5.18	10.00	7.00	8.76	3.82	8.00	4.00
Amateur	10.68	5.30	10.00	7.75	10.25	4.13	10.00	5.25

Here, we report arithmetic mean values, G-iPBI, 26-item version of the German Irrational Performance Beliefs Inventory; G-iPBI-2, 20-item version of the German Irrational Performance Beliefs Inventory; PIB, primary irrational beliefs; LFT, low frustration tolerance; AWF, awfulization; and DEP, depreciation.

#### G-iPBI-2 (20 Items)

Almost identical findings were observed for the 20-item G-iPBI. Again, results indicated that the four dimensions of IB were not multivariate normally distributed (*W* = 0.944, *p* < 0.001). Moreover, the Box M results showed that the group variables performance level and sport-type had similar variance–covariance matrices (*p* > 0.05) in contrast to the gender variable (*p* < 0.05).

##### Age

Results of the correlational analysis showed significant low negative correlations (*r* = −0.16 to −0.33) between athletes’ age and all subscales of the G-iPBI-2.

##### Gender

The three-way MANOVA indicated a significant main effect for gender, *λ* = 0.95, *F*(4,219) = 2.69, *p* = 0.032, *η_p_*^2^ = 0.046. Significant between-subject effects were revealed for all four IB dimensions: PIB, *F*(1,222) = 9.90, *p* < 0.01, *η_p_*^2^ = 0.041, for LFT, *F*(1,222) = 5.57, *p* = 0.023, *η_p_*^2^ = 0.023, for AWF, *F*(1,222) = 20.53, *p* < 0.001, *η_p_*^2^ = 0.079, and for DEP, *F*(1,222) = 11.72, *p* < 0.01, *η_p_*^2^ = 0.052. Table 4 shows that the mean values for all IB dimensions were higher for women than for men.

##### Performance Level

There was no significant main effect for athletes’ performance level, *λ* = 0.94, *F*(8,438) = 1.68, *p* = 0.099, *η_p_*^2^ = 0.029.

##### Sport-Type

Results of the three-way MANOVA indicated a significant main effect for sport-type, *λ* = 0.94, *F*(4,219) = 3.39, *p* = 0.010, *η_p_*^2^ = 0.058. A significant between-subject effect was revealed for PIB, *F*(1,222) = 5.68, *p* = 0.024, *η_p_*^2^ = 0.021 and AWF, *F*(1,222) = 5.33, *p* = 0.025, *η_p_*^2^ = 0.016, both indicating slightly higher values for team athletes (PIB: *M* = 18.82, *SD* = 3.05; AWF: *M* = 17.63, *SD* = 3.38) than for individual athletes (PIB: *M* = 17.86, *SD* = 3.71; AWF: *M* = 16.75, *SD* = 3.88; see lower part of Table 4). There were no significant effects for LFT, *F*(1,222) = 0.08, *p* = 0.771, *η_p_*^2^ < 0.01, and DEP, *F*(1,222) = 2.10, *p* < 0.01, *η_p_*^2^ < 0.01.

In addition, there was a significant interaction effect for performance level and sport-type, *λ* = 0.90, *F*(8,438) = 2.99, *p* < 0.01, *η_p_*^2^ = 0.051. Significant interaction effects were revealed for AWF, *F*(2,222) = 13.54, *p* < 0.01, *η_p_*^2^ = 0.051, and LFT, *F*(2,222) = 8.54, *p* = 0.023, *η_p_*^2^ = 0.042, while no significant effects were obtained for PIB, *F*(2,222) = 2.85, *p* = 0.263, *η_p_*^2^ = 0.02, and DEP, *F*(2,222) = 2.21, *p* = 0.350, *η_p_*^2^ = 0.013. Subsequent *post-hoc* analyses revealed only a significant difference with respect to LFT (*p_bonf_* = 0.01) between semi-elite athletes in team sports (*M* = 16.03, *SD* = 4.69) and amateur athletes in team sport (*M* = 18.62, *SD* = 3.10). Table 5 shows means, and standard deviations separated by performance level and sport-type of the athletes.

## Discussion

The aim of the study was to examine for the first time the relationships between irrational beliefs and important markers of mental health in a German sample of athletes. We included anxiety, depression, and wellbeing as general markers and competition anxiety, perfectionism, and athletic identity as sport-specific markers in our analyses. Since we can only use a reliable and valid measure to capture IBs in athletes, we first undertook a rigorous and detailed translation, cultural adaptation, and validation process to develop a German version of the iPBI. We then performed correlations between the different dimensions of the G-iPBI and aforementioned markers and examined population-based differences.

With regard to the German translation of the iPBI, results of a first CFA indicated a somewhat unacceptable model fit of the data. A closer inspection of the model showed that two of the 28 items had low factor loadings and thus, were removed prior to a second CFA. These include the problematic item already identified by Turner and Allen (2018; see also Nejati et al., 2021), as well as another item of PIB, which was also excluded due to low factor loadings in the original iPBI-2. The 26-item version showed stronger fit indices and an acceptable model fit. In addition, we carried out another CFA on the original 20-item iPBI-2 version, which indicated also a good fit to the data. Moreover, criterion-related validity was established through moderate correlations between respective subscales of the G-iPBI and subscales of an established measure of dysfunctional attitudes. Further results demonstrated the internal stability and the temporal stability of both G-iPBI versions over a period of two weeks, which is consistent with recent research findings (Turner et al., 2018b; Nejati et al., 2021). Taken together, these findings strongly support the four-dimensional structure of the both versions of the G-iPBI as well as their validity and reliability in a German sample of athletes.

Regarding the relationships between IBs and important markers of athletes’ mental health, almost identical findings were observed across the two G-iPBI versions. G-iPBI subscales were positively correlated with athletes’ psychological distress and negatively correlated with wellbeing, implying that the more IBs athletes hold, the higher the frequency of depression and anxiety symptoms and the lower their wellbeing. Results revealed significant low to moderate effect sizes, which is in line with previous studies examining the relationships between IBs and mental health in clinical and non-clinical (Browne et al., 2010; Vîslă et al., 2016) as well as athlete populations (Turner et al., 2018a, 2019a; Chotpitayasunondh and Turner, 2019). Moreover, the G-iPBI showed predominately low correlations to sport-specific competitive anxiety, which underlined the extant literature indicating positive relations between IBs and various forms of anxiety on a cognitive (e.g., speech, social, evaluation, and test anxiety) and physiological level (influence on the individuals’ systolic blood pressure, autonomic physiological arousal, or eating behavior; see for an overview; Turner, 2016). Regarding athletes’ perfectionism, we found similar low correlations with IBs. However, an exception was the moderate correlation between DEP and the Worry subscale of the WAI-T as well as between DEP and the MIPS subscale perfectionistic demands in competitions. This is not surprising, because overgeneralization often occurs in people with social anxiety disorder, as well as those with generalized anxiety, depression, and related conditions, which have very high associations with worry (Beck and Clark, 1997; American Psychiatric Association, 2013). Thus, athletes who have self-doubt or worry about failing under pressure may think they are not good at what they do and feel worthless. Consequently, athletes may feel the need to do everything perfectly in competition in order to receive approval from others and to find self-affirmation that they are not a failure. In summary, we hereby demonstrated that IBs are related to several general and sport-specific markers of mental health, supporting the growing body of research on irrational beliefs and their emotional and behavioral outcomes in athletes.

Concerning the population-based differences, the results showed that women reported higher levels of IBs than men, which is consistent with results from previous studies (Turner and Allen, 2018; Turner et al., 2018a) and generally corresponds to findings from REBT research (Browne et al., 2010). On the one hand, this could be related to the fact that women generally report more mental health problems than men or are more willing to talk about them (Doherty and Kartalova-O’Doherty, 2010). It is therefore not surprising if they also report higher IBs due to existing associations. On the other hand, higher IB in women could also be related to certain social factors or to mental and physical health problems specific to women, as well as to the fact that women as a group are at higher risk of being affected than men. This relates, for example, to biological factors related to reproduction, hormonal changes with the menstrual cycle, biological opportunities during the life cycle, or sex role demands (O’Kelly and Gilson, 2019). Accordingly, such social, psychological, and physical factors could also affect the individuals’ core irrational beliefs. However, the results with respect to the 26-item version must be interpreted with caution, as significance was only marginally warranted. Furthermore, the finding that older athletes experienced less IBs than younger athletes is also in line with previous research (Turner and Allen, 2018, see also Turner et al., 2018a). This relationship may be attributed to, for example, an increase in experience with age, or a change in priority of athletic performance over other aspects of life, or a greater repertoire of strategies for dealing with disturbing thoughts or underlying beliefs (Nicholls and Polman, 2007; Michel-Kröhler et al., 2021).

We also analyzed differences in IBs between amateur, semi-elite, and competitive-elite athletes. We found no significant differences. However, the evidence is mixed, as two studies found significant results between athletes’ performance levels and some dimensions of IBs (Turner and Allen, 2018; Turner et al., 2019b), and another study did not (Nejati et al., 2021). This could be because IBs do not dictate the level of performance. In other words, it is possible to achieve a high level in sports and be irrational at the same time. This could be one reason why psychological problems are becoming more prominent—athletes are able to maintain and develop sporting standards and still hold irrational beliefs. Interestingly, however, we obtained differences in terms of the sport-type, which contradicts the results of Turner et al. (2019b). PIB and AWF were found to be slightly higher in team athletes than in individual athletes. This may be due, for example, to the fact that, in addition to the demands on an athlete to meet or fail certain conditions, appreciation within the team and positive evaluation of one’s contribution to team performance are additional necessities for team athletes, more than for individual athletes. However, this would not be entirely consistent with the results of previous studies showing that individual athletes are more likely to report mental health issues compared to team athletes (Nixdorf et al., 2016). To better understand the relationship between IBs and mental health issues in athletes from different disciplines, future studies should examine the role of IBs in both further cross-sectional but also longitudinal designs. Finally, we found that amateur athletes exhibited lower frustration tolerance compared to semi-elite athletes in team sports, which is consistent with the findings of Turner and Allen (2018), who propose more experience of failure under pressure in semi-elite athletes as an explanation.

### Limitations and Future Research

A first limitation of our study is that the CFI value in terms of model fit indices did not reach the recommended limit of 0.95 (Hu and Bentler, 1999). However, this is not unusual compared to the already established translated versions of the iPBI, which have CFI values between 0.90 and 0.92 (see Urfa and Aşçı, 2018; Chotpitayasunondh and Turner, 2019; an exception is the CFI with a value of 0.96 in the study by Nejati et al., 2021). Beyond that, all other indices are in an acceptable range and correspond to the values of the original measures (Turner and Allen, 2018: SRMR = 0.07, RMSEA = 0.07; Turner et al., 2018a: SRMR = 0.06, RMSEA = 0.07) and the already translated versions (Thai version: Chotpitayasunondh and Turner, 2019: SRMR = 0.07, RMSEA = 0.07; Persian version: Nejati et al., 2021: SRMR = 0.06, RMSEA = 0.06; Turkish version: Urfa and Aşçı, 2018: RMR = 0.06, RMSEA = 0.07). Nevertheless, the G-iPBI should be further investigated in future research to ensure adequate reliability with other and larger samples.

Second, because of the small effect sizes (*η_p_*^2^ < 0.06) and unequal sample sizes (only one-third as many competitive athletes as amateur and semi-competitive athletes), the results of our population-based analyses should not be overestimated. Further studies are needed that, on the one hand, focus on differences between IBs and performance levels, capturing a broader range (e.g., including novice and absolute professional athletes) with approximately equal group sizes to gain deeper insights into IBs in performance development. On the other hand, our study is one of the few that investigates differences in IBs with respect to sport-types. In addition to distinguishing between team and individual athletes, future studies could provide more detailed differentiation in terms of characteristics of specific sport categories (e.g., sports with target focus, aesthetic sports, combat sports etc.) to investigate whether different demands of a particular sport-category are related to more or less IBs.

Finally, we considered the relationships between IBs and social desirability. In contrast to Turner et al. (2018b), we obtained small negative relationships indicating that answering the G-iPBI might be susceptible to response bias. It seems comprehensible that athletes in such a performance-focused environment as competitive sports would switch from rational adaptive beliefs involving “want to” simply to irrational beliefs revolving around “have to” because they think it is a good thing, or rather a necessity, for their athletic career and performance (Botterill, 2005). However, increasing pressure as performance levels rise can also lead athletes to transform their desires into absolute needs (Turner et al., 2019a), for example, to obtain further sponsorship or their squad status. In addition, it cannot be ruled out that some of the athletes’ statements are subject to a certain bias, for example due to memory effects in the form of remembering or forgetting processes, selective perception, or the Hawthrone effect (Döring and Bortz, 2005). Nevertheless, this does not explain why the relationship between IBs and social desirability is negative. Since there are very few studies to date that have examined this relationship (Turner et al., 2018b), future studies should include social desirability in relation with IBs in order to obtain meaningful results.

### Practical Application

Together with the results of previous studies (Turner, 2016; Turner et al., 2022), the findings of the present article provide some indications of how the mental health of athletes can be supported in the context of REBT.

First, since certain subgroups of athletes are more prone to mental health issues than others, this aspect should be considered in the application of REBT interventions. Even though research confirms that REBT is equally effective for men and women (David et al., 2017). O’Kelly and Gilson (2019) recommend going beyond standard REBT when working with woman. The recommendation is based on the fact that certain social factors as well as mental and physical health problems are specific to women (see above in the section “Discussion”). Therefore, in REBT interventions, practitioners should consider identifying, challenging, and changing sex role beliefs (e.g., Heinze et al., 2017) and negative self-evaluations that women tend to hold (for an overview and detailed guidelines see Dryden and Bernard, 2019; and more general guidelines for the use with athletes: Turner and Barker, 2014). In this context, the identification of other factors that influence athletes’ emotions, cognitions, and behaviors, in addition to treating IBs, may be helpful in managing critical performance situations, improving and maintaining athletic performance (Turner, 2016). However, this applies equally to both men and women. Thus, selected sport-specific questionnaires such as the SCRS (Michel-Kröhler et al., 2021) or the WAI-T (Brand et al., 2009) can help to identify competition-limiting factors such as rumination or anxiety in athletes at an early stage in order to initiate specific interventions in addition to REBT or to develop additional strategies, for example, in dealing with disturbing thoughts or debilitating arousal. This could also be interesting when practitioners work with very perfectionistic athletes. These often tend to ruminate during a task, which is exacerbated when the outcome is not as desired. In doing so, athletes become cognitively preoccupied with their inability to achieve perfection or ruminate on past misdeeds and failures (Besser et al., 2004). Perfectionist athletes might therefore benefit from various cognitive interventions that focus on replacing negative repetitive thoughts with positive automatic thoughts (Besser et al., 2004). In addition, another goal in working with perfectionist athletes who are caught up in their PIBs should be to reduce irrational sense of importance (Ellis, 2002), for example, to put a competition into perspective compared to other life events, and thus develop a more constructive and healthier attitude toward their athletic activity.

Second, practitioners should consider the interpretation of irrational and rational beliefs in dependence of athletes’ goals and motivation to achieve their goals. Recent findings (Mesagno et al., 2021) suggest that the influence of irrational (and rational) beliefs on performance is more complex and related to the temporal component of goal attainment. For example, irrational beliefs may increase current sporting endeavor to achieve a short-term goal (e.g., reaching a certain standard at a certain competition). The belief that one must win can be an added incentive to try harder at a particular task, or to focus on what needs to be done to have an advantage over one’s opponent in a competition. In this case, IBs would not be detrimental in the original sense (Turner, 2016; Mesagno et al., 2021) but would have a short-term positive effect on the athlete’s motivation and thus support his or her goal achievement. This could occur especially in the case of athletes at higher performance levels, where the achievement of a goal is associated with further consequences (financial support, sponsorship, inclusion in a selection team, etc.). As a practitioner, however, one must ask to what extent short-term benefits may have long-term negative effects on the athlete’s wellbeing and what is the original, deep-seated motivation of the athlete’s irrational beliefs. It seems relatively unlikely that the maintenance of irrational beliefs benefits athletic performance, in part because of the detrimental effects on mental health (Turner, 2016; Turner et al., 2022). Therefore, a promising approach could be the consideration of IBs together with an athletes’ self-determination (i.e., satisfaction of the three basic needs, namely autonomy, competence, and relatedness). Recent findings (Turner et al., 2022) indicated that irrational beliefs and self-determined motivation operate together as indicators of athletes’ mental health. Irrational beliefs therefore seem more problematic, for example, when motivation for a particular endeavor is regulated in a less autonomous manner, or even when there is a lack of intention to engage. Therefore, the strength of one’s motivation could play an important role in activating IBs. However, the extent to which IBs are problematic for mental health may depend in part on the underlying reasons why the goal is pursued (e.g., intrinsic vs. extrinsic motivated) and the extent to which one feels a sense of autonomy over one’s actions. Thus, in addition to creating an autonomous supportive environment (see for example the TARGET-approach, Braithwaite et al., 2011) in daily sport practice, practitioners might consider how REBT can be used to facilitate autonomous motivation regulation in coaching sessions.

### Conclusion

The current study has presented novel research into the relationship between IBs and important makers of mental health in a German sample of athletes. The validation of a German version of the iPBI contained therein will make it possible in the future to advance research and provide REBT-practitioners in Germany with a diagnostic tool. Since both versions of the G-iPBI do not show notable differences in their psychometric properties, both versions can be validly applied in different performance contexts other than sports (such as education or business). An advantage of the shorter version, besides its comparability with the results of the original English version, is that researchers and practitioners in settings with time-restricted conditions, such as daily training sessions, can apply it more economically. The G-iPBI thus represents a useful, reliable, and ecological measure of performance-related irrational beliefs for future application in research and coaching.

## Data Availability Statement

The datasets presented in this study can be found in online repositories. The names of the repository/repositories and accession number(s) can be found at: https://osf.io/r7eg6/.

## Ethics Statement

The studies involving human participants were reviewed and approved by Local Review Board of the Institute for Psychology of the Johannes Gutenberg-University. Written informed consent from the participants’ legal guardian/next of kin was not required to participate in this study in accordance with the national legislation and the institutional requirements.

## Author Contributions

AM-K collected data of athletes. AM-K and MT performed the statistical analyses and wrote the manuscript. All authors contributed to the article and approved the submitted version.

## Conflict of Interest

The authors declare that the research was conducted in the absence of any commercial or financial relationships that could be construed as a potential conflict of interest.

## Publisher’s Note

All claims expressed in this article are solely those of the authors and do not necessarily represent those of their affiliated organizations, or those of the publisher, the editors and the reviewers. Any product that may be evaluated in this article, or claim that may be made by its manufacturer, is not guaranteed or endorsed by the publisher.

## Footnotes

^1^Model-fit before modification: *N* = 234, χ^2^(164) = 367.09, *p* < 0.001, CFI = 0.908, TLI = 0.894, SRMR = 0.066, RMSEA = 0.076 (90% CI [0.065, 0.086]).
