# Supplementary material for: Link Between Irrational Beliefs and Important Markers of Mental Health in a German Sample of Athletes: Differences Between Gender, Sport-Type, and Performance Level
Source: Front Psychol. 2022 Jul 22;13:918329. doi: 10.3389/fpsyg.2022.918329 (PMC9356198; doi:10.3389/fpsyg.2022.918329)
Supplement: Supplementary file 1 [file Data_Sheet_1.PDF]

Psychological stress and well-being

Results indicated that the three dependent variables (GAD, PHQ, WHO-5) were not multivariate normally distributed ($W = .985$, $p < .001$). In addition, the Box M results showed that the group variables (gender, performance level and sport-type) had similar variance-covariance matrices ($p > .05$).

The three-way MANOVA indicated no significant main effects, either for gender, $\lambda = 1.00$, $F(3,220) = 0.26$, $p = .851$, $\eta p^2 < .01$, performance level, $\lambda = .95$, $F(6,440) = 1.77$, $p = .103$, $\eta p^2 = .023$, or sport-type, $\lambda = .99$, $F(3,220) = 0.52$, $p = .671$, $\eta p^2 < .01$. Table S1a and Table S1b show descriptive values of the respective variables.

Anxiety and perfectionism related to competitions

Results indicated that the five dependent variables (Somatic anxiety, Worry and Concentration difficulties subscales of the WAI-T as well as the Self-related negative reactions to non-perfect performance and Self-related perfectionist demands subscales for the competitive context of the MIPS) were not multivariate normally distributed ($W = .861$, $p < .001$). However, the Box M results showed that the group variables (gender, performance level and sport-type) had similar variance-covariance matrices ($p > .05$).

Gender. Results indicated a significant main effect of athletes' gender on competition related anxiety and perfectionism, $\lambda = .94$, $F(5,218) = 2.51$, $p = .031$, $\eta p^2 = .054$. Separate univariate ANOVAs showed significant between-subject effects for Somatic Anxiety, $F(1,222) = 10.40$, $p = .001$, $\eta p^2 = .037$ and Worry, $F(1,222) = 19.60$, $p < .001$, $\eta p^2 = .077$, indicating that woman experienced significant ($p_{bonf} < .001$) more somatic anxiety ($M = 10.37$, $SD = 3.02$) and worrisome thoughts ($M = 10.44$, $SD = 3.28$) than men (Somatic Anxiety: $M = 8.83$, $SD = 2.90$; Worry: $M = 8.07$, $SD = 2.95$; see Table S1a). In addition, there was a significant between-subject effect for Self-related perfectionistic demands, $F(1,222) = 12.72$, $p < .001$, $\eta p^2 = .045$, indicating significant higher values ($p_{bonf} < .001$) for woman ($M = 27.59$, $SD = 8.67$) compared to men ($M = 23.08$, $SD = 7.48$). No significant differences were obtained for Concentration

difficulties, $F(1,222) = 0.08$, $p = .078$, $\eta p^2 < .01$ and negative reactions to non-perfect performance, $F(1,222) = 0.27$, $p = .610$, $\eta p^2 < .01$. Table S1a showed mean anxiety and perfectionism scores across athletes' gender.

Performance level. The three-way MANOVA indicated no significant main effect for athletes' performance level, $\lambda = .93$, $F(10,436) = 1.69$, $p = .080$, $\eta p^2 = .037$.

Furthermore, there was a significant main effect for the interaction between gender and performance level of athletes, $\lambda = .91$, $F(10,436) = 2.02$, $p = .029$, $\eta p^2 = .044$. However, separate univariate ANOVAs on the outcome variables revealed non-significant interaction effects on Somatic Anxiety, $F(2,222) = 0.41$, $p = .818$, $\eta p^2 < .01$, Worry, $F(2,222) = 3.64$, $p = .195$, $\eta p^2 = .017$, Concentration difficulties, $F(2,222) = 3.04$, $p = .239$, $\eta p^2 = .019$, as well as on Self-related perfectionistic demands, $F(2,222) = 3.21$, $p = .218$, $\eta p^2 = .015$, and Negative reactions to non-perfect performance, $F(2,222) = 1.99$, $p = .388$, $\eta p^2 < .01$, in competitive context (see Table S1b).

Sport-type. Results indicated a significant main effect of athletes' sport-type, $\lambda = .92$, $F(5,218) = 3.53$, $p < .031$, $\eta p^2 = .074$. A significant between-subject effect was revealed for Somatic Anxiety, $F(1,222) = 16.37$, $p < .01$, $\eta p^2 = .079$, and Worry, $F(1,222) = 7.14$, $p = .014$, $\eta p^2 = .029$. Results indicated that individual athletes reported significant higher somatic anxiety ($M = 10.64$, $SD = 3.05$) and worrisome thoughts ($M = 9.87$, $SD = 3.19$) than team athletes (Somatic anxiety: $M = 8.62$, $SD = 2.73$, $p_{bonf} < .001$; Worry: $M = 8.76$ $SD = 3.41$, $p_{bonf} = .01$; see Table 1Sb). In contrast, no significant differences were obtained for Concentration difficulties, $F(1,222) = 3.62$, $p = .064$, $\eta p^2 = .013$, Self-related perfectionistic demands, $F(1,222) = 0.02$, $p = .877$, $\eta p^2 < .01$, and negative reactions to non-perfect performance, $F(1,222) = 0.76$ $p = .388$, $\eta p^2 < .01$.

Table S1a

Effect size differences between subgroups for psychological distress, well-being and competition-related anxiety and perfectionism.

	**Gender**							
	Men (n = 110)				Women (n = 124)			
	M	*SD*	*Md*	*IQR*	*M*	*SD*	*Md*	*IQR*
Psychological distress								
Anxiety	18.48	3.69	18.00	4.00	19.59	3.58	19.00	4.00
Depression	23.33	4.22	22.00	4.75	24.35	4.50	23.00	6.00
Well-being	14.43	4.68	15.00	7.00	13.95	4.28	15.00	6.00
WAI-T								
Somatic Anxiety	8.83	2.90	8.50	4.00	10.37	3.02	10.00	5.00
Worry	8.07	2.95	8.00	4.75	10.44	3.28	10.50	5.00
Concentration difficulties	6.39	2.06	6.00	3.00	6.48	2.34	6.00	3.00
MIPS								
Demands	23.08	7.48	23.00	7.00	27.59	8.67	26.50	12.00
Negative reactions	30.78	10.38	31.00	16.75	32.51	9.97	34.00	15.25

Note. *M* = arithmetic mean values, *SD* = standard deviation, *Md* = median, *IQR* = interquartile range, WAI-T = German version of the Competition Anxiety Inventory, MIPS = Multidimensional Inventory of Perfectionism in Sports, Demands = Self-related perfectionist demands in competitive context, Negative reactions = Self-related negative reactions to non-perfect performance in competitive context.

Table S1b

Effect size differences between subgroups for psychological distress, well-being and competition-related anxiety and perfectionism.

	**Performance Level**												**Sport-type**							
	Competitive-Elite (n = 32)				Semi-Elite (n = 100)				Amateur (n = 102)				Individual (n = 119)				Team (n = 115)			
	M	*SD*	*Md*	*IQR*	*M*	*SD*	*Md*	*IQR*	*M*	*SD*	*Md*	*IQR*	*M*	*SD*	*Md*	*IQR*	*M*	*SD*	*Md*	*IQR*
Psychological distress																				
Anxiety	18.78	3.25	18.00	5.00	18.95	3.71	19.00	4.00	19.27	3.76	18.00	5.50	19.41	3.44	19.00	4.50	18.71	3.87	18.00	4.00
Depression	23.75	3.13	23.50	4.50	23.50	4.71	22.00	5.25	24.27	4.42	23.00	6.00	24.07	4.51	23.00	6.00	23.67	4.28	23.00	6.00
Well-being	13.69	4.48	13.50	7.25	14.51	4.80	15.00	6.25	14.00	4.14	15.00	6.00	13.64	4.29	14.00	7.00	14.73	4.61	15.00	6.00
WAI-T																				
Somatic Anxiety	8.97	2.73	8.00	4.25	9.90	3.31	10.00	5.00	9.61	2.89	9.00	3.75	10.64	3.05	11.00	5.00	8.62	2.73	9.00	4.00
Worry	8.25	2.88	8.00	4.25	9.62	3.67	10.00	5.25	9.38	3.09	9.00	4.75	9.87	3.19	10.00	4.50	8.76	3.41	8.00	5.00
Concentration difficulties	5.62	1.88	5.00	3.00	6.11	2.15	5.00	3.00	7.02	2.24	7.00	3.00	6.58	2.38	6.00	3.00	6.29	2.03	6.00	2.50
MIPS																				
Demands	25.37	6.16	24.50	7.50	24.84	9.65	22.50	12.25	26.12	7.74	25.00	10.00	25.57	8.47	24.00	12.00	25.36	8.41	24.00	10.50
Negative reactions	35.75	8.65	35.00	12.25	30.52	10.42	30.50	16.00	31.58	10.15	32.00	15.00	31.18	9.75	33.00	15.00	32.23	10.62	32.00	15.00

Note. *M* = arithmetic mean values, *SD* = standard deviation, *Md* = median, *IQR* = interquartile range, WAI-T = German version of the Competition Anxiety Inventory, MIPS = Multidimensional Inventory of Perfectionism in Sports, Demands = Self-related perfectionist demands in competitive context, Negative reactions = Self-related negative reactions to non-perfect performance in competitive context.

Table S2

Mean values (M), standard deviations (SD), median values (Md) and interquartile ranges (IQR) for the subscales of the competitive anxiety inventory (WAI-T) and the Multidimensional Inventory of Perfectionism in Sports (MIPS) separated by athletes' gender and performance level.

	Men (n = 110)				Women (n = 124)			
WAI-T subscales	*M*	*SD*	*Md*	*IQR*	*M*	*SD*	*Md*	*IQR*
Somatic anxiety								
Competitive-Elite	8.47	2.36	8.00	3.50	9.41	3.02	9.00	5.00
Semi-Elite	8.74	3.44	8.50	5.00	10.61	3.04	11.00	4.00
Amateur	8.98	2.67	9.00	4.00	10.40	2.99	10.00	5.00
Worry								
Competitive-Elite	7.87	3.00	8.00	5.00	8.59	2.83	9.00	4.00
Semi-Elite	7.60	3.15	7.50	6.00	10.85	3.43	11.50	5.75
Amateur	8.44	2.81	8.00	4.00	10.58	3.03	11.00	5.00
Concentration difficulties								
Competitive-Elite	6.00	2.10	6.00	3.00	5.29	1.65	5.00	2.00
Semi-Elite	6.10	2.20	5.50	3.00	6.11	2.13	5.00	2.00
Amateur	6.68	1.95	7.00	3.00	7.44	2.52	7.00	3.00
MIPS subscales								
Demands								
Competitive-Elite	24.13	5.84	24.00	4.50	26.47	6.40	26.00	10.00
Semi-Elite	20.29	7.99	19.00	11.50	27.63	9.57	25.50	12.75
Amateur	24.67	7.07	23.00	7.00	27.95	8.22	27.00	10.00
Negative Reactions								
Competitive-Elite	35.93	9.63	35.00	8.00	35.59	7.98	34.00	13.00
Semi-Elite	28.79	10.51	29.00	15.50	31.58	10.31	33.50	15.75
Amateur	30.75	10.18	31.00	16.00	32.62	10.13	34.00	13.00

Note. Demands = Self-related perfectionist demands in competitive context, Negative reactions = Self-related negative reactions to non-perfect performance in competitive context.
